# Prognostic Impact of Autophagy Biomarkers for Cutaneous Melanoma

**DOI:** 10.3389/fonc.2016.00236

**Published:** 2016-11-09

**Authors:** Diana Y. L. Tang, Robert A. Ellis, Penny E. Lovat

**Affiliations:** ^1^Dermatological Sciences, Institute of Cellular Medicine, Newcastle University, Newcastle-upon-Tyne, UK; ^2^Dermatology, The James Cook University Hospital, Middlesbrough, UK

**Keywords:** autophagy biomarkers, malignant melanoma, Ambra1, p62, prognostic biomarkers

## Abstract

Prognosis and survival for malignant melanoma is highly dependent on early diagnosis and treatment. While the American Joint Committee on Cancer (AJCC) criterion provides a means of staging melanomas and guiding treatment approaches, it is unable to identify the risk of disease progression of early stage tumors or provide reliable stratification for novel adjuvant therapies. The demand for credible prognostic/companion biomarkers able to identify high-risk melanoma subgroups as well as guide more effective personalized/precision-based therapy is therefore of paramount importance. Autophagy, the principle lysosomal-mediated process for the degradation/recycling of cellular debris, is a hot topic in cancer medicine, and observations of its deregulation in melanoma have brought its potential as a prognostic biomarker to the forefront of current research. Key regulatory proteins, including Atg8/microtubule-associated light chain 3 (LC3) and BECN1 (Beclin 1), have been proposed as potential prognostic biomarkers. However, given the dynamic nature of autophagy, their expression *in vitro* does not translate to their use as a prognostic biomarker for melanoma *in vivo*. We have recently identified the expression levels of Sequestosome1/SQSTM1 (p62) and activating molecule in Beclin 1-regulated autophagy protein 1 (AMBRA1) as novel independent prognostic biomarkers for early stage melanomas. While increasing followed by subsequent decreasing levels of p62 expression reflects the paradoxical role of autophagy in melanoma, expression levels additionally define a novel prognostic biomarker for AJCC stage II tumors. Conversely, loss of AMBRA1 in the epidermis overlying primary melanomas defines a novel prognostic biomarker for AJCC stage I tumors. Collectively, the definition of AMBRA1 and p62 as prognostic biomarkers for early stage melanomas provides novel and accurate means through which to identify tumors at risk of disease progression, facilitating earlier patient therapeutic intervention and stratification tools for novel personalized therapeutic approaches to improve clinical outcome.

Malignant melanoma, the most aggressive form of skin cancer arising from the malignant transformation of melanocytes, is an increasing public health concern worldwide with incidence rates doubling every 10–20 years (1), which now renders this malignancy accountable for 75% of all skin cancer deaths and the most common cause of cancer-related mortality in young individuals between 20 and 35 years of age (2).

As with many cancers, prognosis and survival for melanoma is highly dependent on early detection, diagnosis, and treatment. In line with this need and coupled with the emergence of novel targeted and immunotherapies, current interest is focused on the discovery of predictive and prognostic biomarkers. A biomarker refers to any measurable diagnostic indicator that is used to assess the risk or presence of disease (3). While predictive biomarkers are able to indicate which patient subgroups are likely to benefit from certain treatments (4), prognostic markers enable stratification of patients at initial diagnosis according to eventual outcome, which can be used clinically to guide patient management including the earlier initiation of adjuvant therapies in patients at high risk of disease progression, potentially preventing the development of untreatable metastatic disease (5).

Some of the best established prognostic biomarkers for melanoma are incorporated into the current American Joint Committee on Cancer (AJCC) 2009 staging criterion, the most comprehensive staging system for melanoma to date, which remains the international standard for disease staging and as a guide for treatment approaches. AJCC staging combines several prognostic factors for melanoma, including the depth of invasion (Breslow depth), rate of mitoses, presence of ulceration (loss of the epidermis overlying the tumor), evidence of metastatic spread, and changes in serum lactate dehydrogenase (LDH) to allow risk stratification of morbidity and mortality at the initial diagnosis (6). In general, this divides malignant melanoma into four stages: stages I–II comprising primary tumors of distinct thickness (defined as early stage disease), stage III where locoregional spread of disease (mainly to local lymph node basins) is present, and stage IV where there is presence of distant metastasis.

While early stage melanoma is largely curative by surgical excision, metastatic disease represents the cause of death from melanoma in the vast majority of cases due to a lack of consistently beneficial treatment regimens for late stage disease. Furthermore, despite its comprehensiveness, AJCC staging as a prognostic biomarker is limited by the inability of its criteria to accurately identify high-risk melanoma subgroups that will go on to progress; a particular problem in seemingly “low risk” AJCC stage I melanomas where up to 10% of tumors subsequently metastasize. This emphasizes the urgent need for novel credible biomarkers to identify high-risk tumors as well as the stratification of such patients for more efficacious and earlier therapeutic approaches (6).

Observations of deregulated autophagy in many cancers, including melanoma, have brought this key signaling mechanism to the forefront of much research (7, 8), including its potential capacity as a prognostic biomarker. Autophagy, the principle catabolic process for lysosomal-mediated degradation of intracellular components to sustain cellular energy and survival, is regulated by a complex signaling cascade involving ubiquitin-like conjugation systems, autophagy regulatory proteins [BECN1/Beclin 1 (Beclin-1), activating molecule in Beclin 1-regulated autophagy protein 1 (AMBRA1), Atg8/microtubule-associated light chain 3 LC3 (LC3), and Sequestosome1/SQSTM1 (p62)], and the inactivation of mammalian target of rapamycin (mTOR) to induce activation. Cellular debris within the cytoplasm is sequestered into double-membrane autophagosomes, which are then delivered to lysosomes for degradation and recycling (9). In cancer, however, autophagy plays a paradoxical role; on the one hand, preventing build-up of toxic cellular components that result in genomic stress and instability, thereby promoting tumorigenesis, while, on the other hand, promoting tumor survival of advanced stage solid tumors such as melanoma in a nutrient-deprived hypoxic environment (10). Consistent with the paradoxical role of autophagy and in contrast to observations in benign nevi, electron microscopy studies have shown an increased presence and vacuolization (suggesting degradation) of double-membraned autophagosomes in the cytoplasm of metastatic melanoma cells, thus supporting the notion of increased autophagic activity in advanced stage disease (11, 12).

BRAF is a member of the RAF group of serine/threonine protein kinases, and as such functions to regulate the mitogen-activated protein kinase (MAPK)/extracellular signal-regulated kinase (ERK) cellular growth pathway (13). Activating mutations in BRAF, present and in up to 70% of all melanomas along with NRas mutations (present in approximately 15–20% of melanomas), result in constitutive activation of MAPK signaling, promoting growth, survival, and chemoresistance (14–17). Interestingly, BRAF mutational status in melanoma has also been shown to variably influence autophagy. Following treatment of BRAF wild-type melanoma cells with endoplasmic reticulum (ER) stress-inducing agents *in vitro*, autophagy is activated in line with its pro-survival role; however, when autophagy is inhibited exogenously, this leads to increased cell death (12, 18). Conversely, oncogenic BRAF induces a chronic ER stress status, resulting in enhanced basal autophagy [as evidenced by increased Atg8-PE/LC3-II (LC3-II) expression], resistance of melanoma cells to apoptosis, and insensitivity to further autophagy induction. This suggests that although melanomas rely on increased innate autophagic activity, BRAF-mutated tumors are resistant to further mTOR-dependant stimulation of autophagy and that while combined inhibition of autophagy with chemotherapy might be a viable therapeutic avenue for BRAF wild-type melanomas, targeted therapies that attenuate ER stress may prove a more effective treatment strategy for BRAF mutant melanomas (12, 18, 19).

To date, several markers of autophagy, including LC3 and Beclin 1, have been identified as potential prognostic biomarkers for melanoma. Under normal homeostatic conditions, exogenous LC3 is cytoplasmic but upon autophagy induction becomes conjugated to phosphatidylethanolamine (PE) to form the membrane bound form LC3-II, thus acting as a marker of autophagy induction (20). Increased immunohistochemical expression of LC3 has been shown in malignant melanomas compared to benign nevi (21) and is associated with the development of metastatic disease and poorer outcomes (22). In addition, studies of Beclin 1 expression suggest its’ downregulation parallels melanoma disease stage progression, further supporting a role for autophagy in tumor invasion and metastasis (23, 24). However, although the expression of LC3 provides an indication of autophagy status in melanoma, it is important to note that conversion of LC3-I to LC3-II is a dynamic process, thus limiting the capacity of endogenous LC3 expression as an accurate biomarker of autophagy status and importantly, the reflection of autophagic flux (15). Moreover, although reported to be downregulated in melanoma, there is also conflicting evidence of Beclin 1 overexpression in advanced melanoma, thus questioning its expression as a reliable prognostic biomarker (25).

Sequestosome1/SQSTM1 is a scaffold protein that shuttles ubiquitinated proteins into the autophagosome, later degraded along with other autophagosomal contents upon fusion with a lysosome. Impairment of autophagy is therefore reflected by an associated accumulation of p62, a process reported to be a key to the onset of tumorigenesis (17). Conversely, decreased levels of p62 reflect active autophagy, as observed in advanced stage melanomas where autophagy is commonly reactivated to enhance tumor survival (Figure 1A). Data from our lab have further defined p62 expression as a prognostic biomarker for melanoma where a stepwise increase in expression is observed in early AJCC stage melanomas (increased above basal levels in benign nevi and reflecting deregulated autophagy) but which is subsequently decreased in advanced metastatic tumors, consistent with the reactivation of autophagy and its paradoxical role in cancer [Figures 1B,C; (26)]. Furthermore, univariate analysis showed a significantly increased risk of metastasis in AJCC stage II tumors with low p62 expression (<20% median p62 expression) compared to those with high expression (>20% p62 expression) [Figure 1D; (26)]. Moreover, since there was no association with Breslow depth or tumor ulceration, p62 expression defines an independent stratifying variable from AJCC staging prognosticators. Collectively, these data highlight p62 as a novel independent prognostic biomarker for AJCC stage II melanomas, providing a powerful tool for refining the risk of disease progression and enabling earlier patient therapeutic intervention. In addition, p62 expression may represent a companion biomarker of response to autophagy modulation *in vivo*, an important concept in view of emerging autophagy modulator therapies (27) and their potential to improve overall clinical outcome for patients with metastatic melanoma.

**Figure 1:**
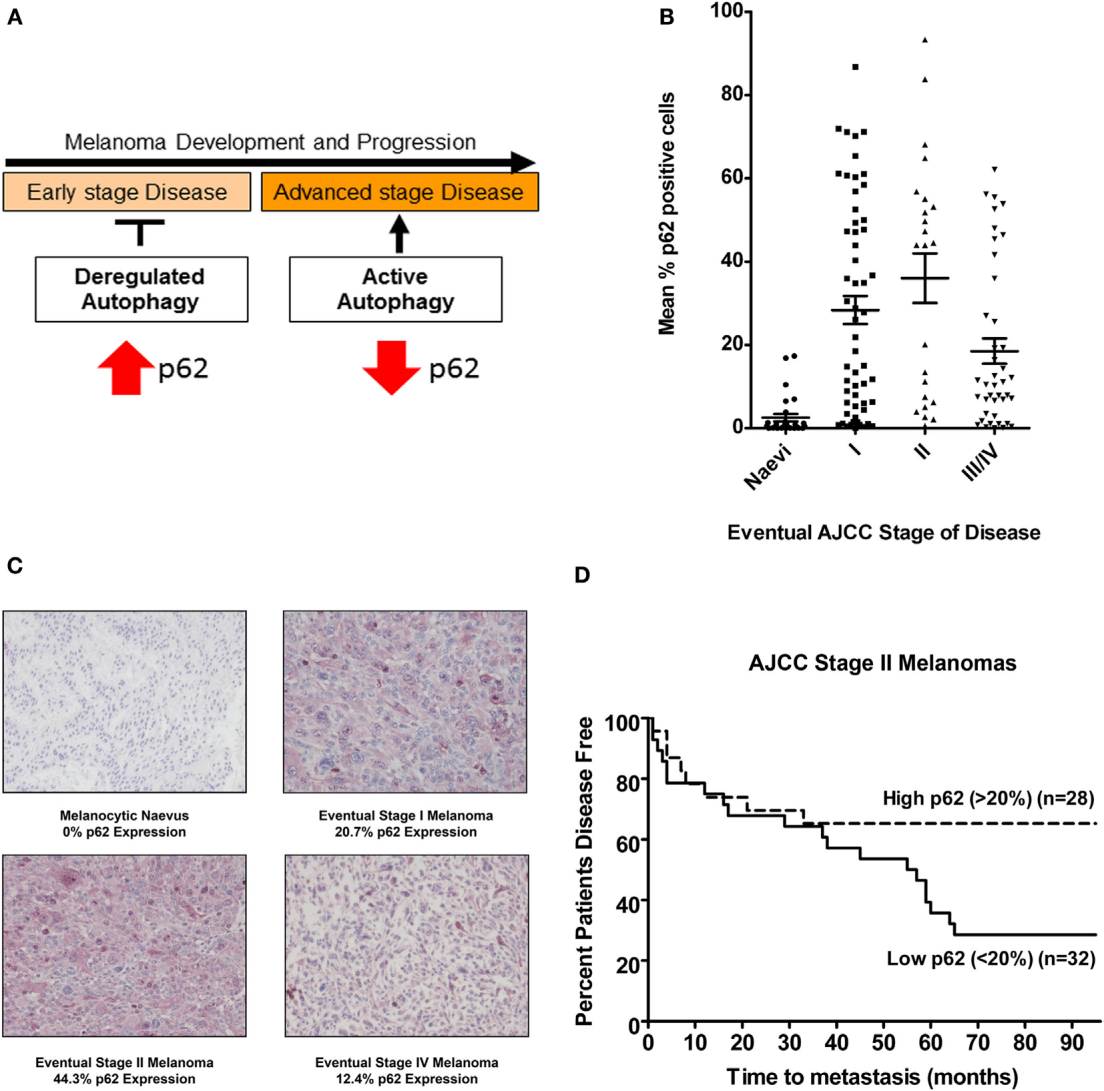
**p62 expression is a prognostic biomarker for AJCC stage II melanomas**. **(A)** Schematic of the paradoxical role of autophagy in melanoma in the context of p62 expression; impairment of autophagy drives tumorigenesis of early stage melanomas reflected by p62 accumulation, whereas decreased levels of p62 seen in advanced disease reflect autophagy reactivation. **(B)** Mean % p62 expression in a cohort of eventual AJCC stage I, II, III, and IV melanomas or benign nevi after a minimum 5-year follow-up. Each point represents the mean % of p62 positive cells. Horizontal lines representing median p62 expression levels indicate an increase in median p62 expression levels between benign nevi and AJCC stage I or II melanomas and a relative decrease in expression in advanced AJCC stages III and IV tumors (Kruskal–Wallis *P* < 0.0001) (26). **(C)** Photomicrographs of immunohistochemical p62 expression and mean % in a melanocytic nevus or an eventual AJCC stage I, II, or IV melanoma. Scale bar = 100 μm. **(D)** Univariate analysis of mean p62 expression in AJCC stage II primary tumors demonstrating an increased risk of metastasis in tumors expressing >20% p62 [Log-Rank (Mantel–Cox) *P* = 0.031, HR 2.29 (95% CI 1.08–4.86)], and highlighting the potential of p62 as a prognostic biomarker (26).

Activating molecule in Beclin 1-regulated protein 1 (AMBRA1) is a component of the Beclin 1/VPS34 complex and involved in the formation of PI3K rich membranes during the nucleation phase of autophagy [Figure 2A; (28)]. As a key autophagy initiating regulatory protein, AMBRA1 represents a potential marker of autophagy induction as well as a possible therapeutic target for autophagy inhibition. However, in addition to its functional role in autophagy, a growing body of evidence supports a role for AMBRA1 in cellular differentiation (29, 30) including in the early differentiation of neuronal stem cells in which autophagy is activated to fulfill the high energy demands of this process (28, 31). In line with these findings, we have recently demonstrated the role of AMBRA1 in epidermal differentiation with the expression *in vivo* increasing in line with keratinocyte differentiation from the basal layer of the epidermis to the uppermost layer, the *stratum corneum* (32). Unlike p62, however, the expression of AMBRA1 in primary melanomas is variable and as such its value as a tumoral biomarker remains undefined. Strikingly, however, our recent data demonstrate the decreased or even complete loss of AMBRA1 expression in the epidermis overlying many AJCC stage I melanomas (Figure 2B), which did not correlate with the degree of epidermal invasion and was not observed in benign nevi (32). These data suggest that the expression of AMBRA1 in the melanoma microenvironment may have prognostic potential. Univariate analysis of an initial cohort of 129 all AJCC stage melanomas further revealed decreased or loss of epidermal AMBRA1 expression was significantly associated with decreased disease-free survival, with stratification for AJCC stage I disease, additionally revealing epidermal AMBRA1 expression as a putative biomarker of disease progression [Figure 2C; (32)]. Again, there was no correlation with Breslow depth suggesting, such as p62, that epidermal AMBRA1 expression is also a biologically distinct marker for AJCC stage I melanomas. This is a striking finding considering that these tumors are normally regarded as low risk, with currently no alternative means of identifying specific individuals whose tumors are likely to progress.

**Figure 2:**
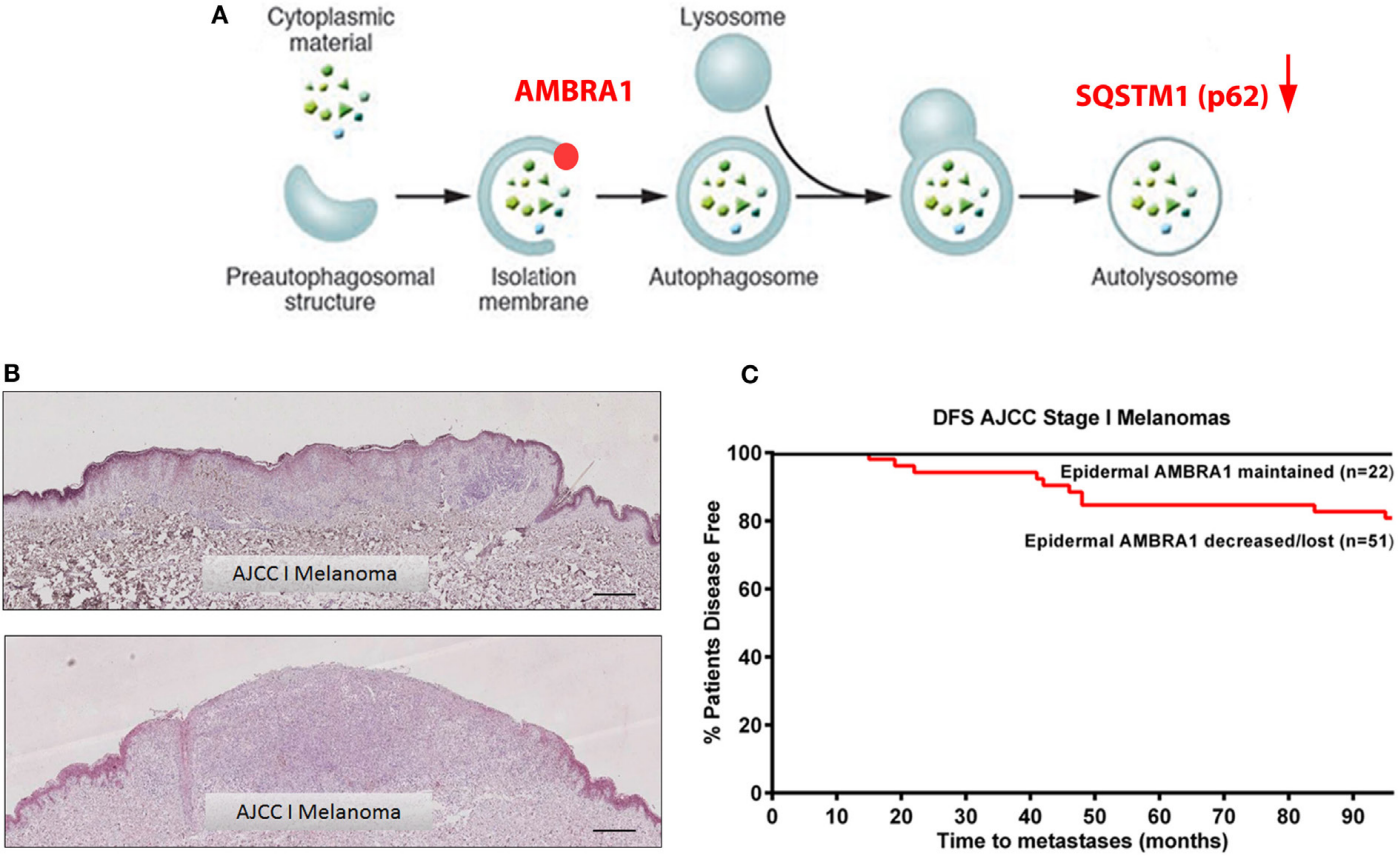
**Loss of epidermal AMBRA1 identifies a high-risk AJCC stage I melanoma subgroup**. **(A)** Schematic representation of the autophagy pathway highlighting the role of AMBRA1 in the nucleation phase of autophagy and indicating interplay of p62. **(B)** Representative immunohistochemistry images of epidermal AMBRA1 expression depicting maintained (top image) or loss of AMBRA1 expression (bottom image) in the epidermis overlying AJCC stage I melanomas. Loss of epidermal AMBRA1 expression overlying the tumor tissue creates a “watershed” area, where the epidermis distant to the tumor reveals a normal pattern of AMBRA1 expression. Scale bar = 100 μm. **(C)** Kaplan–Meier curve showing decreased 7-year disease-free survival in 51 AJCC stage I tumors where epidermal AMBRA1 was decreased or lost as compared with 22 tumors where AMBRA1 expression was maintained [Log-Rank (Mantel–Cox) test *P* < 0.03, HR 4.3 (95% CI 1.14–16.51)]. Epidermal AMBRA1 expression of each tumor was recorded as being either maintained, decreased, or lost based on the perceived degree of loss of epidermal AMBRA 1 expression overlying the tumor bulk compared to normal epidermis within the same section.

Since the current and universally adopted AJCC staging system is unable to identify the risk of disease progression in seemingly “low risk” early stage melanomas, such tumors are only identified after the onset of metastatic disease progression, at which point treatment options are limited and frequently ineffective. Critically, identifying, refining, and validating prognostic biomarkers for early stage melanomas such as the proposed biomarkers of autophagy will thus enable the identification of high-risk tumor subgroups. Both p62 and AMBRA1 expression exemplify how autophagy can be harnessed as prognostic biomarkers for melanoma, each providing clinically relevant information over and above AJCC staging, which in particular will be useful for refining the risk of melanoma progression in patients with AJCC stage I or II melanomas. Ultimately, further validation of these biomarkers will allow application in a clinical context, facilitating both earlier therapeutic intervention and the refinement of personalized therapies for malignant melanoma to improve clinical outcome and the prevention of premature loss of life.

## Author Contributions

DT: main author of the paper and corresponding author involved in all aspects of authorship. RE: substantial contributions to the acquisition, analysis, and interpretation of data for the work including production of figures for the paper. PL: senior author, contribution to the analysis of data and the writing of the manuscript; accountable for all aspects of the work. All the authors have undertaken final approval of the final manuscript version to be published.

## Conflict of Interest Statement

The authors declare that the research was conducted in the absence of any commercial or financial relationships that could be construed as a potential conflict of interest.
